# Chorionic villus-derived mesenchymal stem cell-mediated autophagy promotes the proliferation and invasiveness of trophoblasts under hypoxia by activating the JAK2/STAT3 signalling pathway

**DOI:** 10.1186/s13578-021-00681-7

**Published:** 2021-10-13

**Authors:** Yijing Chu, Chengzhan Zhu, Chongyu Yue, Wei Peng, Weiping Chen, Guifang He, Changchang Liu, Yang Lv, Guoqiang Gao, Ke Yao, Rendong Han, Xiaoyu Hu, Yan Zhang, Yuanhua Ye

**Affiliations:** 1 Department of Obstetrics and Gynaecology, The Affiliated Hospital of Qingdao University, 16 Jiangsu Road, Qingdao, 266000 China; 2 Medical Animal Laboratory, The Affiliated Hospital of Qingdao University, Qingdao, China; 3 Department of Hepatobiliary and Pancreatic Surgery, The Affiliated Hospital of Qingdao University, Qingdao, China

**Keywords:** Chorionic villous-derived mesenchymal stem cells, Trophoblasts, Autophagy, JAK2/STAT3 signalling pathway

## Abstract

**Background:**

Trophoblast dysfunction during pregnancy is fundamentally involved in preeclampsia. Several studies have revealed that human chorionic villous mesenchymal stem cells (CV-MSCs) could regulate trophoblasts function.

**Results:**

To understand how human chorionic villous mesenchymal stem cells (CV-MSCs) regulate trophoblast function, we treated trophoblasts with CV-MSC supernatant under hypoxic conditions. Treatment markedly enhanced proliferation and invasion and augmented autophagy. Transcriptome and pathway analyses of trophoblasts before and after treatment revealed JAK2/STAT3 signalling as an upstream regulator. In addition, STAT3 mRNA and protein levels increased during CV-MSC treatment. Consistent with these findings, JAK2/STAT3 signalling inhibition reduced the autophagy, survival and invasion of trophoblasts, even in the presence of CV-MSCs, and blocking autophagy did not affect STAT3 activation in trophoblasts treated with CV-MSCs. Importantly, STAT3 overexpression increased autophagy levels in trophoblasts; thus, it positively regulated autophagy in hypoxic trophoblasts. Human placental explants also proved our findings by showing that STAT3 was activated and that LC3B-II levels were increased by CV-MSC treatment.

**Conclusion:**

In summary, our data suggest that CV-MSC-dependent JAK2/STAT3 signalling activation is a prerequisite for autophagy upregulation in trophoblasts.

**Graphic abstract:**

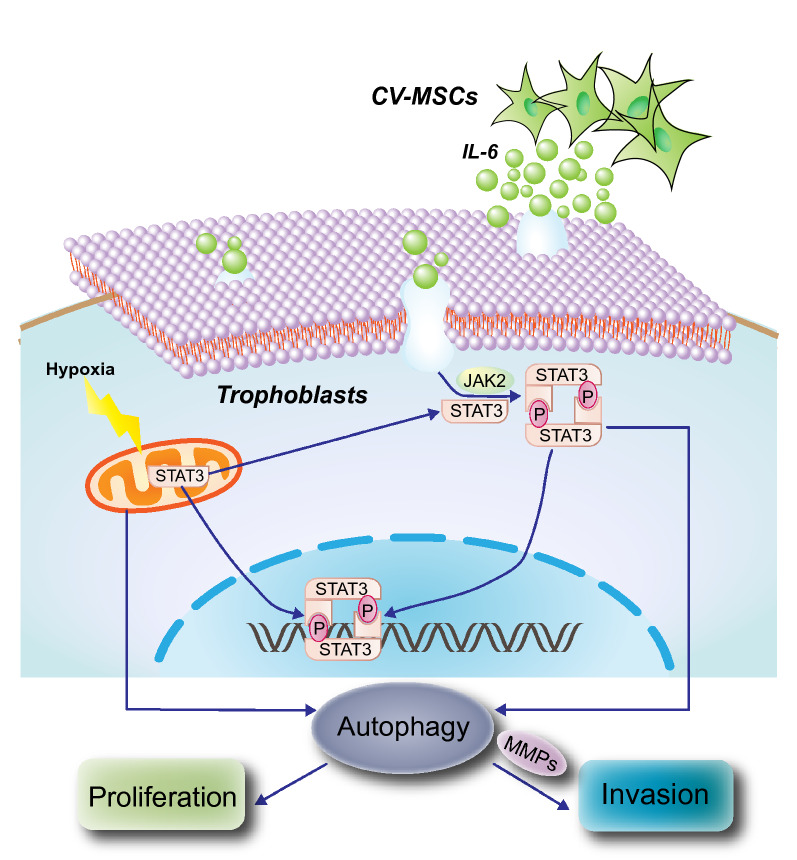

**Supplementary Information:**

The online version contains supplementary material available at 10.1186/s13578-021-00681-7.

## Introduction

As a special kind of hypertensive disorder occurring during pregnancy, preeclampsia (PE) is one of the primary causes of maternal and perinatal morbidity and mortality [1–3]. Although increasing numbers of researchers have worked to understand the molecular mechanisms of PE progression, the underlying mechanisms responsible for its pathogenesis and the inducing factors of PE remain elusive. It has been suggested that the proliferative and invasive capacity of extravillous trophoblasts (EVTs) is essential for placenta development; however, failed spiral artery remodelling and insufficient oxygen and nutrient supply cause EVT invasion dysfunction, which ultimately results in placental dysfunction and adverse pregnancy outcomes [4–6]. In addition, aberrant placental implantation can cause a state of increased oxidative stress and hypoxia, which lead to inflammation and antiangiogenic protein release [7].

Since autophagy degrades aggregates of damaged organelles or misfolded proteins and manipulates the “regeneration” of cells, it is a vital process for cell survival under stress and inflammation [8, 9]. Due to the hypoxic and inflammatory responses associated with PE, placental trophoblasts in individuals with PE are more reliant on autophagy for survival than normal cells [10, 11]. Autophagy protects syncytiotrophoblasts from apoptosis, infection, and inflammation in the human placenta [12, 13], but many questions remain regarding the exact aetiology and precise pathogenic mechanisms ensuring autophagic flux.

Chorionic villi, which form the innermost layer of the placenta and are critical components of the maternal-foetal interface, continue growing throughout pregnancy to enrich the foetus with nutrients and blood supply from the mother [5, 14]. Placental pathologies, such as PE and foetal growth restriction, are related to the dysplasia or dysfunction of placental chorionic villi; thus, they are also crucial for placental development [4, 15]. Foetal CV-MSCs are crucial for normal placental development; as multipotent stromal cells, CV-MSCs are detached from chorionic villi [16]. Many studies have suggested that CV-MSCs can promote angiogenesis through paracrine effects and potentially participate in placental pathologies in the vascular system, including PE as well as foetal growth restriction. However, there is currently no information on the roles of CV-MSCs in the proliferative and invasive capacities of EVTs.

Here, the effects of CV-MSC conditioned medium (CM) on the proliferative and invasive capacities of the trophoblast lines JAR, JEG-3 and HTR-8 were studied. In addition to increasing the proliferative and invasive capacities, CV-MSC CM significantly enhanced autophagy in the three trophoblast lines under hypoxic conditions. Transcriptome analyses showed considerable upregulation of the inflammatory response and IL-6/JAK/signal transducer and activator of transcription 3 (STAT3) signalling pathway in trophoblasts; JAK2/STAT3 was putatively recognized as a positive upstream regulator of autophagy under hypoxic conditions.

## Methods and materials

### Cell culture

Placentas were obtained from full‑term births after caesarean section (n = 2) and age-matched placentas from severe preeclampsia after caesarean section (n = 2) with parental permission. All CV-MSCs were used at passages 3–6 in this study. Every procedure was conducted in accordance with the ethical protocols of the Affiliated Hospital of Qingdao University, China. In brief, the foetal portion of the placenta was minced into approximately 1-mm^3^ slices and then rinsed with PBS to remove all blood. The tissues were treated with trypsin (0.25%) and collagenase (0.1%) (type I; Sigma-Aldrich, St. Louis, MO). Afterward, they were incubated at 37 °C for 30 min. After filtration through a 100-μm nylon filter and centrifugation, the cells were plated on culture plates in stem cell culture medium (SCCM), which contained Stem Cell Basic Medium (Dakewe Biotech Co., Guangzhou, China) and 5% UltraGRO™ (Helios, USA). An incubator with a temperature of 37 °C and 5% CO_2_ was used to culture the primary cells.

The JAR, JEG-3, and HTR-8 cells obtained from the Type Culture Collection of China Centre were subjected to culture and then used for experiments. DMEM/F12 containing 10% FBS was used to culture all three trophoblast lines in an incubator at 37 °C and 5% CO_2_. The medium was changed when the confluency reached 50%. The cells were subjected to incubation for a set time at 37 °C, and the humidified atmosphere of the incubator contained 93% N_2,_ 5% CO_2,_ and 2% O_2_ (Invivo2 Hypoxia Workstation, Ruskinn Technology, Leeds, West Yorkshire, UK). For each experiment, the cells were subjected to culture in triplicate.

### CV-MSC identification

Flow cytometry (with antibodies obtained from eBioscience, San Diego, CA, including CD34, CD105, CD73, CD90, CD44, CD45, IG1 and HLA-DR) was used to examine the expression of cell markers in CV-MSCs (passage 3); these markers included positive markers (CD44, CD73, CD90 and CD105) and negative markers (CD34, CD45, CD146, IG1 and HLA-DR).

Moreover, CV-MSCs from normal placenta or severe preeclampsia placenta can change into osteoblasts as well as adipocytes by differentiation; therefore, we assessed this ability. CV-MSCs cultured in 6-well plates were grown to approximately 70–80% confluence. Then, the CV-MSCs were cultured in differentiation medium (osteogenic or adipogenic) (Gibco, Carlsbad, CA) for 3 weeks. Alizarin red S was used to stain the CV-MSCs to verify osteoblast differentiation; for adipocyte differentiation, oil red O was selected.

### CM preparation

When CV-MSCs isolated from the placentas reached 80% confluence, we replaced the medium with DMEM/F12 (Gibco, Carlsbad, CA) without FBS and cultured the cells for an additional 24 h. After a 12-min centrifugation (1200×*g*), the medium was passed through a filter (0.22 µm) (Millipore, Billerica, MA). Then, it was collected for subsequent experiments. DMEM/F12 medium without FBS was used as control CM.

### Antibodies and reagents

We purchased anti-microtubule-associated protein LC3 and BECN1 (beclin1, an autophagosome initiator) antibodies from Sigma-Aldrich Corporation (St. Louis, USA). Cell Signaling Technology (Danvers, MA, USA) provided the following antibodies: anti-STAT3, anti-pSTAT3, anti-JAK2, anti-pJAK2, anti-P62, anti-MMP2, and anti-MMP9. Cryptotanshinone (a STAT3 inhibitor), 3-methyladenine (an autophagy inhibitor) (3-MA, 10 mM) and bafilomycin A1 (Baf A1, an autophagosome-lysosome fusion inhibitor) were purchased from MedChem Express (NH, USA). A STAT3 plasmid was purchased from Shanghai Genechem Co., Ltd. Recombinant human interleukin-6 (IL-6) and IL-6 antibodies were purchased from R&D Systems.

### RNA-seq and gene set enrichment analysis (GSEA)

TRIzol Reagent (1 ml) (Thermo Fisher Scientific) was used to extract the samples; these samples were then stored at − 80 °C. We prepared libraries based on the Illumina TruSeq RNA Sample Prep Kit instructions and carried out sequencing on a MiSeq instrument. RSEM software was used to analyse the experimental data from Annoroad Gene Technology Co., Ltd. (Beijing, China). These data were labelled as SRR9943697-SRR9943702 (RNA-seq) and can be accessed at the Sequence Read Archive (SRA).

c2.cp.v5.1.symbols from the gene set database was used to carry out GSEA of the RNA-seq data after fold change data input into GSEA software (Broad Institute) to analyse differential expression.

### Transmission electron microscopy

PBS (7.4 pH) with glutaraldehyde (2.5%) was used to fix the trophoblasts, which were then incubated at 4 °C for more than 2 h. OsO4 (1%) was used to post-fix the cells for 2 h; then, they were dehydrated in ethyl alcohol and propanone. Next, the cells were imbedded in epoxy resin. Ultrathin sections (50–60 nm) were cut and placed on uncoated copper grids; 3% lead citrate-uranyl acetate was used to stain these ultrathin sections. A JEM-1200EX transmission electron microscope was used to obtain images (JEOL, Tokyo, Japan).

### Quantitative real-time PCR (RT-PCR)

CV-MSC CM was used to treat JAR, JEG-3, and HTR-8 cells for 24 h. Subsequently, we extracted total RNA from the trophoblasts using TRIzol Reagent (Thermo Fisher Scientific). Then, we used a reverse transcription kit (Invitrogen) to synthesize complementary DNA. Gene-specific TaqMan probes (Applied Biosystems) and Master Mix (Thermo Fisher Scientific) were used to carry out quantitative RT-PCR according to the instructions of the manufacturer. The expression of each target gene was normalized to GAPDH expression. We used TaqMan probes for IL6 (Hs00985639_m1), STAT3 (Hs00374280_m1), GAPDH (Hs02786624_g1), and leukaemia inhibitory factor (LIF; Hs01055668_m1), and three separate reactions were performed for each marker.

### Trophoblast invasion assay

Cells from the three trophoblast lines (5 × 10^5^ cells) were seeded in Transwell chambers in 24-well plates (Corning, NY, USA). Fifty microliters of a 1:10 dilution of Matrigel™ was used to cover the pore membranes (8 μm) in twenty-four-well plates. The trophoblasts were cultured on the membranes for 12 h. One of two types of medium was added to the lower chamber: CV-MSC CM supplemented with 10% FBS or normal culture medium supplemented with 10% FBS (control). Transwell assays were performed while culturing the trophoblasts without FBS for 24 h. Next, 4% paraformaldehyde was used to fix all cells. Trophoblasts that penetrated through the membranes were analysed. The cells that migrated through the membrane were counted in high-magnification fields, and every experiment was performed three times.

### Cell proliferation analysis

We added cells from the three trophoblast lines to ninety-six-well plates (density: 5000 cells per well), cultured these cells, and measured trophoblast proliferation daily via CCK-8 assays (Thermo Fisher Scientific, MA, USA). We added CCK-8 reagent to each well, and the trophoblasts were cultured for an additional 1.5 h. Then, colourimetric assays were performed by measuring the absorbance (optical density [OD] value) of each well in a microplate reader (wavelength: 450 nm). Growth curves were determined in three independent experiments.

### Western blotting

Trophoblasts were lysed on ice for 12 min using RIPA buffer (Sigma, St. Louis, MO, USA). After centrifugation at 12,000×*g*, the cell lysates were treated with LDS sample buffer. SDS-PAGE was used to separate the protein mixtures, which were then transferred from the gel to a polyvinylidene fluoride (PVDF) membrane (Bio-Rad, Hercules, CA). Next, 5% skim milk was used to block the membrane.

Subsequently, primary rabbit monoclonal antibodies against human JAK2, pJAK2, STAT3, pSTAT3, MMP2, MMP9, LC3, BECN1 and P62 (1:1000 dilution) or β-actin (same dilution; Proteintech, Chicago, IL) were incubated with the blocked membranes. Then, secondary antibodies were incubated with the membranes (1:1000; CST, Danvers, MA). We detected and quantified the protein-antibody complexes using a chemiluminescence detection system (Bio-Rad, Hercules, CA).

### Placental explant culture

Within a 10-min operation, all placentas were collected, treated within 30 min and closely examined for any visible abnormalities. After thorough rinsing with PBS 3 times to remove the maternal blood, the placental villous tissues were chopped into 8-mm^3^ pieces (2 mm × 2 mm × 2 mm). DMEM/F12 (4 ml per well) with 1% penicillin/streptomycin and amphotericin B (Gibco, Carlsbad, CA) was used to culture the placental explants in six-well dishes (Corning) in a hypoxic incubator for 48 h at 37 °C with 2% oxygen. After CV-MSC CM treatment for 24 h, PBS was used to rinse the explants; after that, they were frozen in liquid nitrogen.

### Immunohistochemistry

Paraformaldehyde (4%) was used to fix the human term placental explants (n = 5) for 60 min. We embedded the tissues in paraffin, sliced them into 4-μm sections, and deparaffinized them. Then, the slides were boiled in 6.0 pH sodium citrate buffer (10 mM) for 7 min at 120 °C for antigen retrieval. Hydrogen peroxide was used to block endogenous peroxidase for 10 min. We subsequently washed the slides three times for 5 min each with TBS, which contained 0.05% Tween 20 (TBS/T; Merck; Darmstadt, Germany); later, these slides were incubated with monoclonal anti-STAT3 antibodies (1:200) and anti-P62 antibodies (1:1000) for 12 h at 4 °C. Diluted biotinylated secondary antibodies were incubated with the sections for twenty min at 37 °C. We visualized the target proteins via fresh DAB solution and used haematoxylin as a tissue counterstain. Through an optical microscope (Olympus FV500, Tokyo, Japan), the expression of the target proteins was assessed by two observers independently. The area and intensity of staining in five random regions (200× magnification) were analysed with Image-Pro Plus 5.1 to assess the protein expression levels.

### Statistical analysis

One-way analysis of variance (ANOVA) or two-tailed Student’s t-test was used to carry out statistical analyses; the data are reported as the mean ± standard deviation from more than three experiments performed independently. If the P value was less than 0.05, it indicated a significant difference.

## Results

### Growth, invasion, and autophagy were promoted in trophoblasts by CV-MSCs under hypoxic conditions

**Fig. 1:**
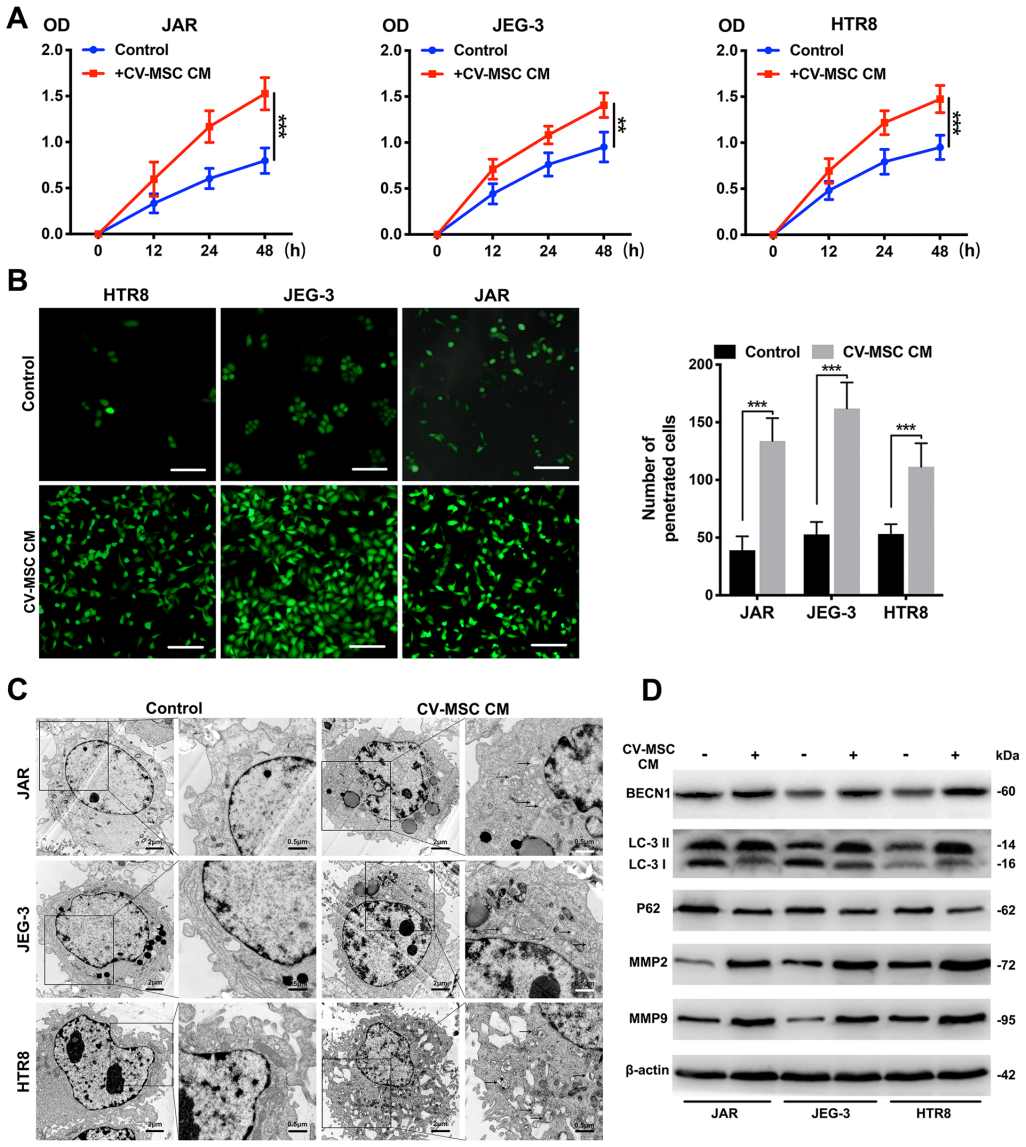
CV-MSCs promote the autophagy, proliferation and invasion of trophoblasts in vitro. **A** Representative CCK-8 assay results for JAR, JEG-3 and HTR-8 cells are shown. Trophoblast cells were treated with CV-MSC CM under hypoxic conditions. **B** Representative Transwell assay results for trophoblasts are shown. The number of trophoblasts that migrated through the 8-μm Transwell membrane pores was counted to determine changes in the invasive capabilities in response to CV-MSC CM under hypoxic conditions (scale bar, 50 μm). **C** Transmission electron microscopy (TEM) analysis showed autophagosomes in the cytoplasm of trophoblast cells treated with CV-MSC CM under hypoxic conditions (scale bar, 1 μm). **D** Whole cell lysates from trophoblast cells were subjected to western blotting to analyse LC3, BECN1, p62, MMP2 and MMP9 levels. β-actin was included as a loading control. ***P < 0.001

First, we isolated CV-MSCs from healthy and severe preeclampsia placentas and identified their multidirectional differentiation ability and surface marker expression (Additional file 1: Fig. S1A, B). Next, we used CCK-8 assays, Transwell assays and transmission electron microscopy to examine the proliferation, invasion, and autophagy of trophoblasts treated with or without CV-MSC CM under hypoxic conditions (Fig. 1A–C). The CV-MSC CM-treated trophoblasts exhibited significantly higher proliferation than the untreated cells under hypoxic conditions (all P < 0.001). Based on the Transwell assay results, CV-MSC CM increased the number of penetrated trophoblasts after 24 h of exposure to the hypoxic culture system (P < 0.001). To verify whether CV-MSCs from severe PE placentas have the same trophoblast-promoting effects, we examined the proliferation and invasion of trophoblasts treated with CM from PE-CV-MSCs. The PE-CV-MSC CM-treated JEG-3 and HTR-8 trophoblasts exhibited slightly higher proliferation than the untreated cells (all P > 0.05), and the invasion ability of the three trophoblast lines was not increased after treatment with PE-CV-MSC CM (Additional file 2: Fig. S2A, B). These results indicated that the function of CV-MSCs in PE placenta may be impaired by pathological conditions.

To investigate the effects of CV-MSCs on trophoblast autophagy, an electron microscopy analysis was performed, and the results revealed that there were more autophagosomes in CV-MSC CM-treated trophoblasts than in control trophoblasts (Fig. 1C). In accordance with these observations, western blotting indicated that BECN1 expression and the LC3-II/LC3-I ratio increased, while the p62 level decreased in the three trophoblast lines after CV-MSC CM treatment under hypoxic conditions (Fig. 1D). Matrix metalloproteinases (MMPs), a group of zinc-dependent endopeptidases, play crucial roles in the promotion of cell growth and metastasis [17]. Many research had confirmed the relationship between MMP2/9 and trophoblasts invasion [18, 19]. Increased MMP2 and MMP9 levels were also observed in cells treated with CV-MSC CM (Fig. 1D). Thus, CV-MSCs promoted trophoblast proliferation, invasion, and autophagy.

### Global transcriptome characterization of trophoblasts regulated by CV-MSCs under hypoxic conditions

**Fig. 2:**
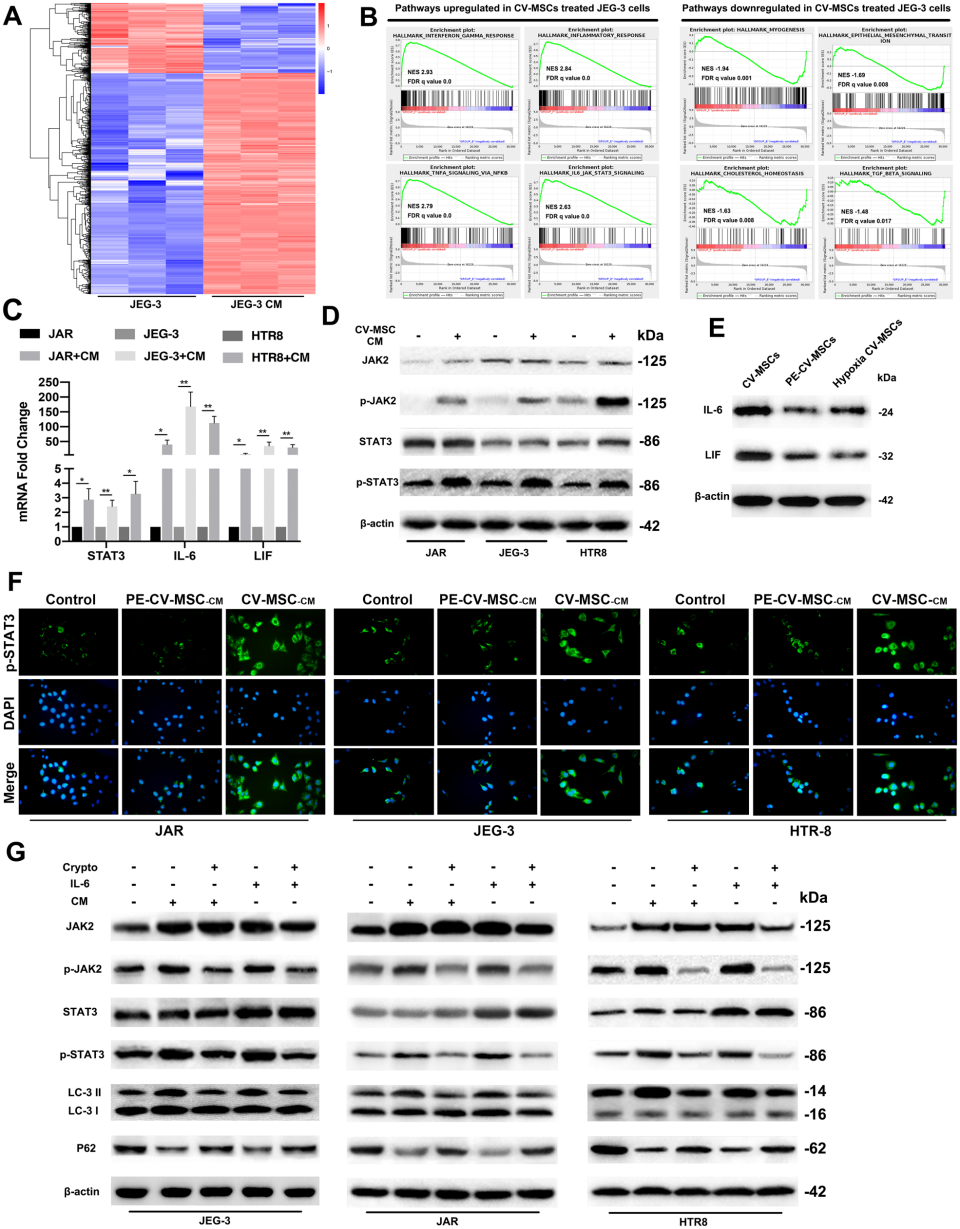
Global transcriptome characterization of trophoblasts regulated by CV-MSCs; JAK2/STAT3 signalling pathway activation by CV-MSCs mediates increased trophoblast autophagy. **A** Heat map of some differentially expressed genes in JEG-3 cells treated with or without CV-MSC CM under hypoxic conditions according to RNA-seq. **B** GSEA of the most upregulated and downregulated pathways in JEG-3 cells treated with CV-MSC CM compared with JEG-3 cells cultured alone. **C** Significant increases in STAT3, IL-6 and LIF mRNA levels were found in JAR, JEG-3 and HTR-8 cells treated with CV-MSC CM by qRT-PCR. **D** Significant increases in p-JAK2 and p-STAT3 were found in JAR, JEG-3 and HTR8 cells treated with CV-MSC CM under hypoxic conditions by western blotting. **E** Immunoblot analysis of IL-6 and LIF protein levels in normal CV-MSCs, PE-CV-MSCs and CV-MSCs under hypoxia condition. **F** Three trophoblast cell lines were cultured with normal CV-MSC CM or PE CV-MSC CM, and p-STAT3 expression was tested by Immunofluorescence. **G** Three trophoblast cell lines were cultured under hypoxic conditions with or without cryptotanshinone, IL-6 or CV-MSC CM, and JAK2, p-JAK2, STAT3, p-STAT3, LC3 and P62 expression levels were tested by western blotting analysis. The results are expressed as the means ± SD. ***P < 0.001

To better understand the changes in JEG-3 cells that occurred after CV-MSC treatment, we compared the transcriptomes of JEG-3 cells cultured for 48 h with or without CV-MSC CM under hypoxic conditions. Through RNA-seq, we found 336 upregulated and 859 downregulated genes that were changed more than two-fold (Fig. 2A). Moreover, GSEA was used to compare the signalling pathway changes between CV-MSC CM-treated trophoblasts and control trophoblasts. From the results, cytokine signalling pathway upregulation was proven, and JAK2/STAT3 signalling was identified as one of the pathways that showed the most significant upregulation in CV-MSC CM-treated trophoblasts (Fig. 2B). In summary, our analyses revealed that CV-MSCs upregulated the proliferation, invasion, and autophagy of trophoblast cells, and IL-6/JAK/STAT3 signalling was upregulated significantly in this process.

### The JAK2/STAT3 signalling pathway was activated in trophoblasts cultured with CV-MSC CM

We further examined how CV-MSC CM treatment influenced the JAK2/STAT3 signalling pathway in trophoblasts under hypoxic conditions. CV-MSC CM-treated trophoblasts exhibited approximately threefold higher STAT3 mRNA levels than untreated trophoblasts after 48 h (Fig. 2C). IL-6 and LIF, which are STAT3 activators, were dramatically upregulated in CV-MSC CM-treated trophoblasts compared with untreated trophoblasts (Fig. 2C). Consistent with the mRNA data, JAK2, STAT3, pJAK2, and pSTAT3 protein levels were significantly increased in CV-MSC CM-treated trophoblasts compared to control trophoblasts (Fig. 2D). To evaluate the JAK2/STAT3 signalling pathway and autophagy in PE-CV-MSC CM-treated trophoblasts under hypoxic conditions, we examined the mRNA and protein expression of STAT3 and autophagy-related markers (Additional file 2: Fig. S2C, D). There was no significant STAT3 signalling activation or increased autophagy activity in trophoblasts after PE-CV-MSC CM treatment, and the IL-6 and LIF mRNA levels were stable in PE-CV-MSC CM-treated trophoblasts compared with untreated trophoblasts.

Since we performed all the experiments under hypoxic conditions, we then examined JAK2/STAT3 signalling, autophagy and MMPs expression in trophoblasts cultured under normal oxygen and hypoxic conditions. Our results showed that hypoxia slightly increased autophagy activity and MMP2/MMP9 expression, and the JAK2/STAT3 signalling pathway factors were not activated in trophoblasts (Additional file 3: Fig. S3A). As shown in Fig. 2E, we determined the IL-6 and LIF protein levels in normal CV-MSCs, PE-CV-MSCs and CV-MSCs under hypoxia condition using western blotting assays. To further compare the STAT3 activation effects of normal CV-MSCs and PE-CV-MSCs on trophoblasts, we examined the p-STAT3 levels in three lines trophoblasts treated with PBS, CV-MSC-CM and PE-CV-MSC-CM using immunofluorescence staining. The results confirmed that the PE-CV-MSCs had a lower effect of STAT3 inactivation in trophoblasts than normal CV-MSCs (Fig. 2F).

These results reveal that the JAK2/STAT3 signalling pathway could be activated by CV-MSC treatment in trophoblasts under hypoxic conditions, and CV-MSCs in healthy donor placentas exhibit higher activity than those in PE patient placentas.

### The JAK2/STAT3 signalling pathway mediated increased trophoblast autophagy under hypoxic conditions

After confirming that CV-MSCs activated autophagy through the JAK2/STAT3 signalling pathway in trophoblasts, we investigated whether this signalling pathway regulated trophoblast autophagy. Trophoblasts were stimulated with the STAT3 activator IL-6 and the specific STAT3 inhibitor cryptotanshinone. As a bioactive compound, cryptotanshinone is extracted from the roots of *Salvia miltiorrhiza* Bunge, and it blocks STAT3 phosphorylation at Tyr705 [20]. The p-STAT3/STAT3 ratio in JEG-3 cells was almost fifty percent lower when 8 mM cryptotanshinone was present than when it was absent (Fig. S3B). Additionally, the expression of autophagy-related proteins in CV-MSC CM-treated cells was investigated in the presence of cryptotanshinone (8 mM). We discovered that p-STAT3, p-JAK2 and BECN1 levels and the LC3-II/LC3-I ratio decreased; moreover, p62 levels in the three trophoblast lines were higher in the presence of 8 mM cryptotanshinone than in its absence (Fig. 2G). We studied the same parameters in cells treated with cryptotanshinone (8 mM) and IL-6 (Fig. 2G).

To determine whether the IL-6 present in CV-MSC CM is responsible for the observed effects, we examined JAK2/STAT3 signalling and autophagy-related proteins in trophoblasts treated with an IL-6 antibody and CV-MSC CM using western blotting (Additional file 3: Fig. S3C). The results showed that IL-6 neutralization by CM by antibodies attenuated the CV-MSC CM-induced activation of STAT3 signalling and autophagy in trophoblasts. Thus, the results showed that STAT3 acted as a positive regulator of CV-MSC-induced autophagy and that IL-6 secreted by CV-MSCs may act as a STAT3 signalling activator in trophoblasts.

### CV-MSCs promoted trophoblast growth and metastasis through the JAK2/STAT3 signalling pathway

**Fig. 3:**
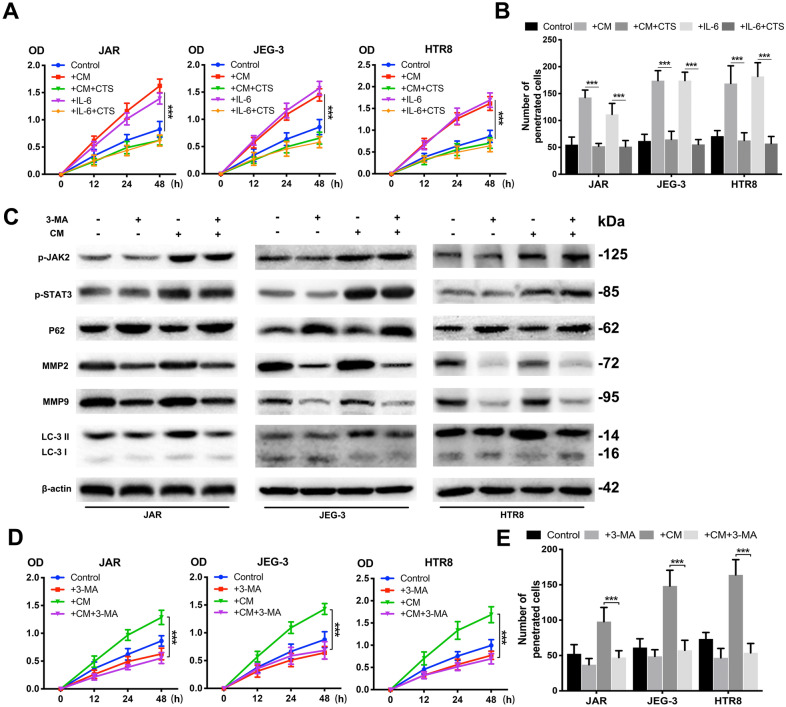
CV-MSCs promoted trophoblast growth and metastasis through the JAK2/STAT3 signalling pathway. **A** Cell proliferation was evaluated by a CCK-8 assay. The three trophoblast cell lines were treated with IL-6 or CV-MSC CM and with or without cryptotanshinone under hypoxic conditions. Trophoblast cells cultured alone under hypoxic conditions served as controls. **B** Trophoblast cells were treated with IL-6 or CV-MSC CM and with or without cryptotanshinone, and the invasion abilities were determined by Transwell assay. The number of penetrated cells was counted and analysed statistically. **C** The three trophoblast cell lines were cultured under hypoxic conditions with or without 3-MA or CV-MSC CM, and p-JAK2, p-STAT3, MMP2, MMP9 and LC3 expression levels were tested by western blotting analysis. **D** Cell proliferation was evaluated by a CCK-8 assay. The three trophoblast cell lines were treated with 3-MA or CV-MSC CM under hypoxic conditions. Trophoblast cells cultured alone under hypoxic conditions served as controls. **E** Trophoblast cells were treated with 3-MA or CV-MSC CM under hypoxic conditions, and invasion abilities were determined by Transwell assay. The number of penetrated cells was counted and analysed statistically. ***P < 0.001

To further investigate how the JAK2/STAT3 signalling pathway influenced trophoblast regulation by CV-MSCs, we analysed trophoblast proliferation and invasion after cryptotanshinone treatment. Cells were cultured in CV-MSC CM or IL-6 for 24 h; then, cryptotanshinone (8 mM) was used to culture them for 24 h. A CCK-8 assay was used to investigate the changes in cell growth (Fig. 3A). IL-6 and CV-MSC CM individually increased cell viability, while cryptotanshinone reversed the enhancing effects of IL-6 and CV-MSC CM. Transwell assays were also performed, and we found the same trends in cells when cryptotanshinone and IL-6 or CV-MSC CM was added (Fig. 3B). These results demonstrated that CV-MSCs accelerated trophoblast proliferation as well as invasion through the JAK2/STAT3 signalling pathway.

### Autophagy inhibition decreased trophoblast growth and invasion even under conditions of JAK2/STAT3 signalling pathway activation

To assess whether autophagy inhibition is connected to decreased growth and invasion in trophoblasts, we treated trophoblasts cultured with or without CV-MSC CM for 24 h with an autophagy inhibitor (3-MA, 10 nM). The western blot results confirmed that 3-MA reduced the LC3-II/LC3-I ratio as well as MMP2 and MMP9 levels in trophoblasts, even in CV-MSC CM-treated trophoblasts (Fig. 3C). pJAK2 and pSTAT3 expression levels were increased in the three trophoblast lines treated with CV-MSC CM and with or without 3-MA (Fig. 3C). Then, the proliferation and invasion abilities of trophoblasts treated with 3-MA and CV-MSC CM were tested using CCK-8 and Transwell assays. Even in the presence of CV-MSC CM, 3-MA suppressed the growth and invasion of the three trophoblast lines (Fig. 3D, E).

To further determine the effect of CV-MSCs on the autophagic flux of trophoblasts, BECN1 and P62 levels and the LC-3 II/LC3-I ratio were compared in trophoblasts treated with CV-MSC CM in the presence of bafilomycin A1 (Baf A1) (Additional file 3: Fig. S3D). The LC-3 II/LC3-I ratio and BECN1 and P62 levels were significantly increased in trophoblasts treated with Baf A1. In contrast, the LC-3 II/LC3-I ratio was not significantly different between Baf A1-treated and untreated cells when they were cotreated with CV-MSC CM. Moreover, the BECN1 and P62 levels were increased significantly in Baf A1-treated and untreated cells when they were cotreated with CV-MSC CM. These results indicated that CV-MSC CM may activate trophoblast autophagy by inducing autophagic flux, and inhibiting autophagy can inhibit the proliferation and invasion abilities of trophoblasts with or without CV-MSC CM treatment.

### STAT3 regulated autophagy in trophoblasts under hypoxic conditions

**Fig. 4:**
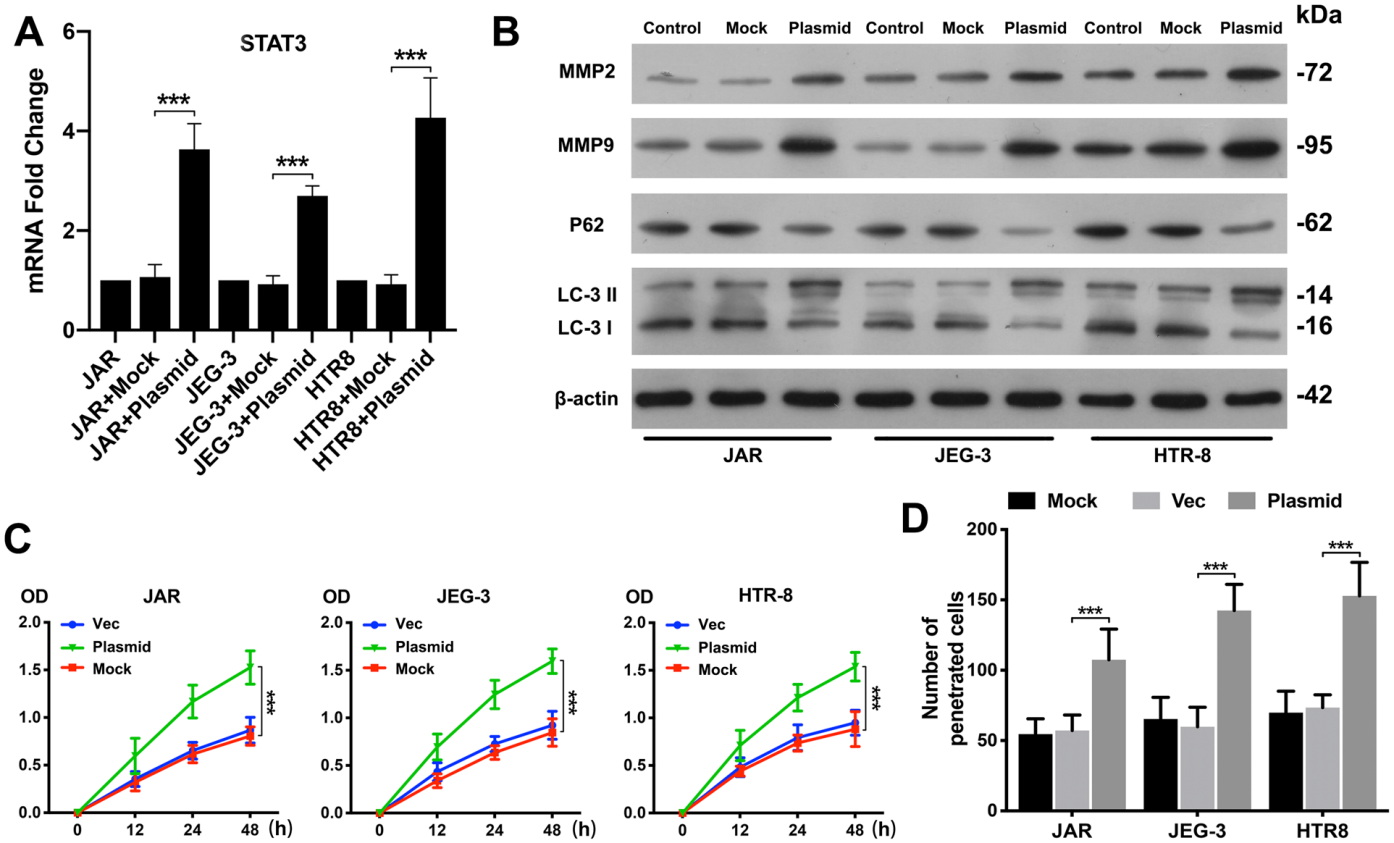
STAT3 regulates autophagy in trophoblast cells under hypoxic conditions. **A** Significant increases in STAT3 mRNA were found in JAR, JEG-3 and HTR-8 cells transfected with a STAT3 overexpression plasmid compared to untreated cells (Mock) or empty vector (Vec)-transfected cells under hypoxic conditions by qRT-PCR. **B** The three trophoblast cell lines were transfected with a STAT3 overexpression plasmid under hypoxic conditions, and MMP2, MMP9, P62 and LC3 expression levels were tested by western blotting analysis. Untreated trophoblast cells (Mock) and empty vector (Vec) cells served as controls. **C** Cell proliferation was evaluated by a CCK-8 assay. The three trophoblast cell lines were transfected with a STAT3 overexpression plasmid or empty vector under hypoxic conditions. Trophoblast cells cultured alone served as controls. **D** Trophoblast cells were transfected with a STAT3 overexpression plasmid or empty vector under hypoxic conditions, and invasion abilities were determined by Transwell assay. The number of penetrated cells was counted and analysed statistically. *P < 0.05, **P < 0.01, ***P < 0.001

To examine whether STAT3 activation increases trophoblast autophagy, viability, and invasion, we transfected trophoblasts with a STAT3 overexpression plasmid and examined STAT3 mRNA expression in trophoblasts (Fig. 4A, P < 0.001). A CCK-8 assay indicated that cell viability was significantly increased by STAT3 plasmid transfection compared to empty control vector transfection (Fig. 4C, P  < 0.001). Consistent with the CCK-8 assay results, the Transwell assay results suggested that STAT3-overexpressing trophoblasts showed higher invasive ability than control trophoblasts, as determined by the number of penetrated cells (Fig. 4D, P  < 0.001).

We further examined autophagy-related protein expression and MMP2/MMP9 levels in STAT3 plasmid-transfected trophoblasts (Fig. 4B). The western blotting results showed that P62 levels were decreased in these cells, whereas the MMP2/MMP9 and LC3 II levels were enhanced, suggesting that STAT3 positively regulates trophoblast autophagy.

### CV-MSC-mediated STAT3 activation increased autophagy in placental explants

**Fig. 5:**
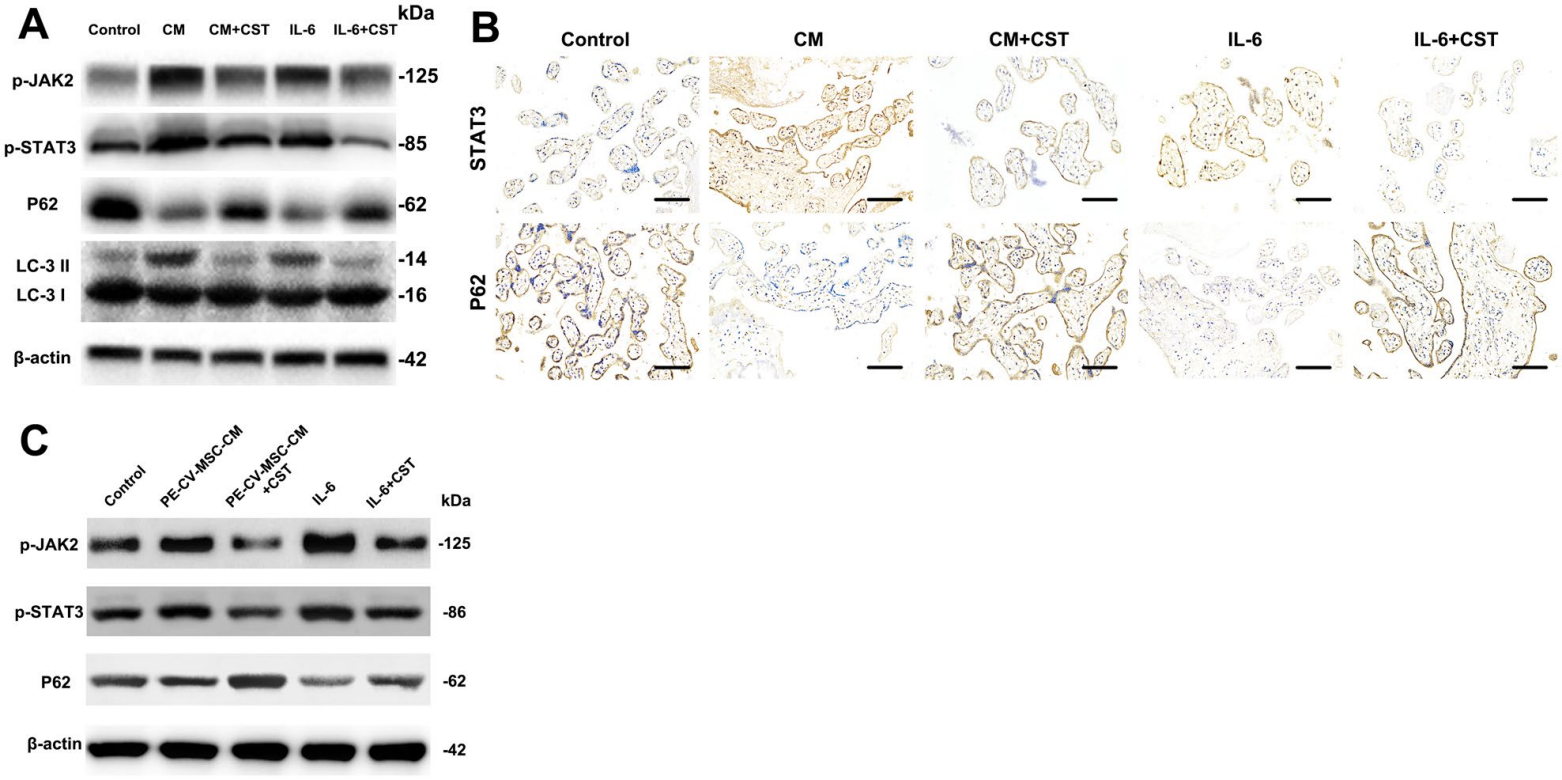
CV-MSC-mediated STAT3 activation increased autophagy in placental explants under hypoxic conditions. **A** Placental explants were treated with IL-6 or CV-MSC CM and with or without cryptotanshinone under hypoxic conditions, and p-JAK2, p-STAT3, P62 and LC3 expression levels were tested by western blotting analysis. Untreated placental explants under hypoxic conditions served as controls. **B** Placental explants were treated with IL-6 or CV-MSC CM and with or without cryptotanshinone under hypoxic conditions, and STAT3 and P62 expression levels were tested by immunohistochemistry. Untreated placental explants served as controls (scale bar, 50 μm). **C** Placental explants were treated with IL-6 or PE-CV-MSC CM and with or without cryptotanshinone under hypoxic conditions, and p-JAK2, p-STAT3, P62 and LC3 expression levels were tested by western blotting. Compared with **A**, PE-CV-MSC CM showed a lower effect of STAT3 and autophagy activation

Next, the data from trophoblasts were compared with placental explant cultures, which are in vivo models in which villous trophoblasts remain in a natural environment under more physiologically relevant conditions than traditional cell culture. The human placental explants were incubated in DMEM/F12 and then in medium containing CV-MSC CM or IL-6 with or without the STAT3 inhibitor cryptotanshinone for 48 h. Western blot assays were used to determine the p-JAK2, pSTAT3, and LC3 II levels in the placental explants. While P62 levels were decreased in the CV-MSC CM- and IL-6-treated placental explants compared to the control explants, p-JAK2, pSTAT3, and LC3 II levels showed obvious increasing trends (Fig. 5A). Additionally, immunohistochemistry showed increased STAT3 staining intensity in the villous cytotrophoblast cytoplasm and decreased P62 staining intensity in the villous cytotrophoblast cytoplasm in CV-MSC CM-treated placental explants (Fig. 5B) compared to control explants. Interestingly, syncytiotrophoblast nuclei remained unstained in the cryptotanshinone-treated group, suggesting that CV-MSCs activate STAT3 and enhance villous cytotrophoblast autophagy. Furthermore, we found that the PE-MSCs showed a lower effect of STAT3 and autophagy activation on placental explant compared to the normal CV-MSCs (Fig. 5C).

Taken together, these data suggest that CV-MSCs mediate JAK2/STAT3 activation and autophagic activity in cultured placental explants.

## Discussion

It is vital that autophagy be regulated precisely in placentation, and some evidence suggests that autophagy in the placenta is impaired during PE [21]. Shigeru Saito et al. proposed an autophagy inhibition hypothesis for the pathophysiology of PE: at the early stage of pregnancy, abnormal placentation is induced by autophagy inhibition in trophoblasts; thus, poor placentation leads to chronic and severe hypoxia in the placenta. Furthermore, severe hypoxia in the placenta exaggerates trophoblast impairment as well as inflammation and homeostasis dysregulation, resulting in a vicious cycle [22]. Although autophagy status changes are observed in pre-eclamptic placentas, the mechanisms that modulate autophagy and autophagy-related biological changes in placentas remain unknown. Here, we provide evidence that CV-MSC-mediated JAK2/STAT3 signalling pathway activation is the regulatory mechanism of autophagic activity in human villous trophoblasts and that it promotes trophoblast proliferation and invasion.

Many studies have proven the therapeutic potential of mesenchymal stem cells (MSCs), multipotent cells isolated from various human tissues, in tissue repair; this therapeutic potential is attributed to the capacity of these cells to undergo multipotent differentiation and modulate immune responses [23–25]. CV-MSCs that are capable of self-renewal and differentiation and that exhibit immunoregulatory properties can be isolated from human term placentas, which are abundant and commonly discarded after delivery [26]. Some studies have revealed roles of CV-MSCs in the regulation of endothelial cells, macrophages, natural killer cells, and dendritic cells [27–30]. Due to the outstanding properties of CV-MSCs, they are potential alternative MSCs that can be applied in cell-based therapy [31], but research on the functions of these cells has been limited. We investigated how human CV-MSCs influenced trophoblasts under hypoxic conditions and found that CV-MSCs regulate autophagy to influence trophoblast proliferation and invasion abilities through the JAK2/STAT3 signalling pathway.

As a potential transcription factor, STAT3 acts as a mediator of the interactions between extracellular signalling molecules (e.g., cytokines and growth factors) and polypeptide receptors on the surface of cells [32]. STAT3 is maintained at a basal level in cells, whereas it sharply increases via self-activation if stimulation occurs. In addition, STAT3 is activated constitutively in a majority of malignant tumours with high invasiveness and autophagy levels, suggesting that STAT3 works as an oncogene [33]. Recent literature has also explored how STAT3 regulates autophagy and has emphasized that STAT3 affects autophagy in various ways [34]. Some studies have indicated that STAT3 seems to inhibit autophagy in some types of tumours and infectious diseases [35, 36], but others have shown that STAT3 activation can significantly enhance autophagy in some blood tumours and autoimmune and inflammatory diseases [37, 38]. STAT3, which responds to a variety of autophagy inducers, is a well-known stress-responsive nuclear factor [34]. Several studies have revealed that the STAT3 may be involved in the pathophysiologic processes of preeclampsia [39–41]. However, it is still not completely understood how specific stimuli drive STAT3 signalling and how it regulates autophagy.

Trophoblast dysfunction, which leads to cellular ischaemia and hypoxia, oxidative stress and vascular endothelial injury, is involved in hypertensive diseases during pregnancy [42]. Current PE treatment strategies focus on improving placental microcirculation and preventing maternal and foetal complications [3]. However, there is no treatment addressing the aetiology of trophoblastic dysfunction despite the clear importance of trophoblast autophagy in placentation and PE. It has been described in previous studies that autophagy, a defence mechanism in healthy cells, is driven by p53 during human trophoblast differentiation [43]. Autophagy activation has been shown to be efficacious in helping to prevent normal cell dysfunction and as a targeted therapeutic strategy in multiple preclinical models; for example, the activation of autophagy protects hepatocytes from chemical-induced hepatotoxicity [22]. These findings could provide novel insight into potential PE therapies.

Because the symptoms of PE usually occur after 20 weeks of gestation, the condition is first observed long after the failure of trophoblast invasion (usually complete before 8–10 weeks of gestation). Furthermore, our results emphasize the urgent need for the early diagnosis and treatment of placental abnormalities. However, it remains unclear how CV-MSCs influence other types of placenta-based cells or even in vivo; thus, it remains to be studied whether CV-MSCs affect them similarly. Further study on how STAT3 regulates autophagy will be important for thorough insight into PE physiology and for developing a novel therapeutic strategy for PE, such as CV-MSC treatment.

## Conclusion

In summary, it was described in our study that CV-MSCs promote the proliferation and invasion of trophoblasts under hypoxic conditions in vitro, at least partially due to JAK2/STAT3 signalling activation, which upregulates trophoblast autophagy. Our findings provide new potential therapeutic or preventive approaches for placenta-related disorders during pregnancy.

## Supplementary Information

**Additional file 1: Figure S1.** Characterization of primary CV-MSCs derived from human placental tissues. (A) Representative photomicrographs of primary human CV-MSCs and PE-CV-MSCs before confluence at passage 3. The cells were examined for osteogenic and adipogenic differentiation. Scale bar = 20 μm. (B) The purity of the isolated CV-MSCs and PE-CV-MSCs was examined by flow cytometry; CV-MSCs express CD44, CD73, CD90 and CD105, but lack CD34, CD45, CD146, IG1 and HLA-DR expression.

**Additional file 2: Figure S2.** Effect of PE-CV-MSC CM on the proliferation, invasion, autophagy and JAK2/STAT3 signalling of trophoblasts. (A) Representative CCK-8 assay results in JAR, JEG-3 and HTR-8 cells are shown. Trophoblast cells were treated with PE-CV-MSC CM under hypoxic conditions. (B) The number of trophoblasts that migrated through the 8-μm Transwell membrane pores was counted to determine changes in the invasive capabilities in response to CV-MSC CM under hypoxic conditions. (C) STAT3, IL-6 and LIF mRNA levels were determined in JAR, JEG-3 and HTR-8 cells treated with PE-CV-MSC CM by qRT-PCR. (D) Protein expression of p-JAK2, p-STAT3, LC3 II, BECN1, P62 and MMP2/9 was examined in JAR, JEG-3 and HTR-8 cells treated with PE-CV-MSC CM by western blotting.

**Additional file 3: Figure S3.** Effect of hypoxia, IL-6 antibody and Baf A1 on trophoblasts treated with CV-MSC CM. (A) Three trophoblast cell lines were cultured under normal oxygen or hypoxic conditions, and p-JAK2, p-STAT3, BECN1, P62, LC3 and MMP2/9 expression levels was tested by western blotting analysis. (B) STAT3 in JEG-3 cells treated with cryptotanshinone at the indicated concentration or DMSO as a control for 60 min before culture in growth medium were tested by western blotting. (C) IL-6 antibodies were added to trophoblast cells treated with or without CV-MSC CM under hypoxic conditions, and p-JAK2, p-STAT3, BECN1, P62, LC3 and MMP2/9 expression levels were tested by western blotting analysis. Untreated trophoblast cells served as a control. (D) Baf A1 was added to trophoblast cells treated with or without CV-MSC CM under hypoxic conditions, and the expression of BECN1, P62 and LC3 was tested by western blotting analysis. Untreated trophoblast cells served as a control.

## Data Availability

The datasets used and/or analyzed during the current study are available from the corresponding author on reasonable request.
