# Supplementary figures and images for: Chorionic villus-derived mesenchymal stem cell-mediated autophagy promotes the proliferation and invasiveness of trophoblasts under hypoxia by activating the JAK2/STAT3 signalling pathway
Source: Cell Biosci. 2021 Oct 13;11:182. doi: 10.1186/s13578-021-00681-7 (PMC8513187; doi:10.1186/s13578-021-00681-7)

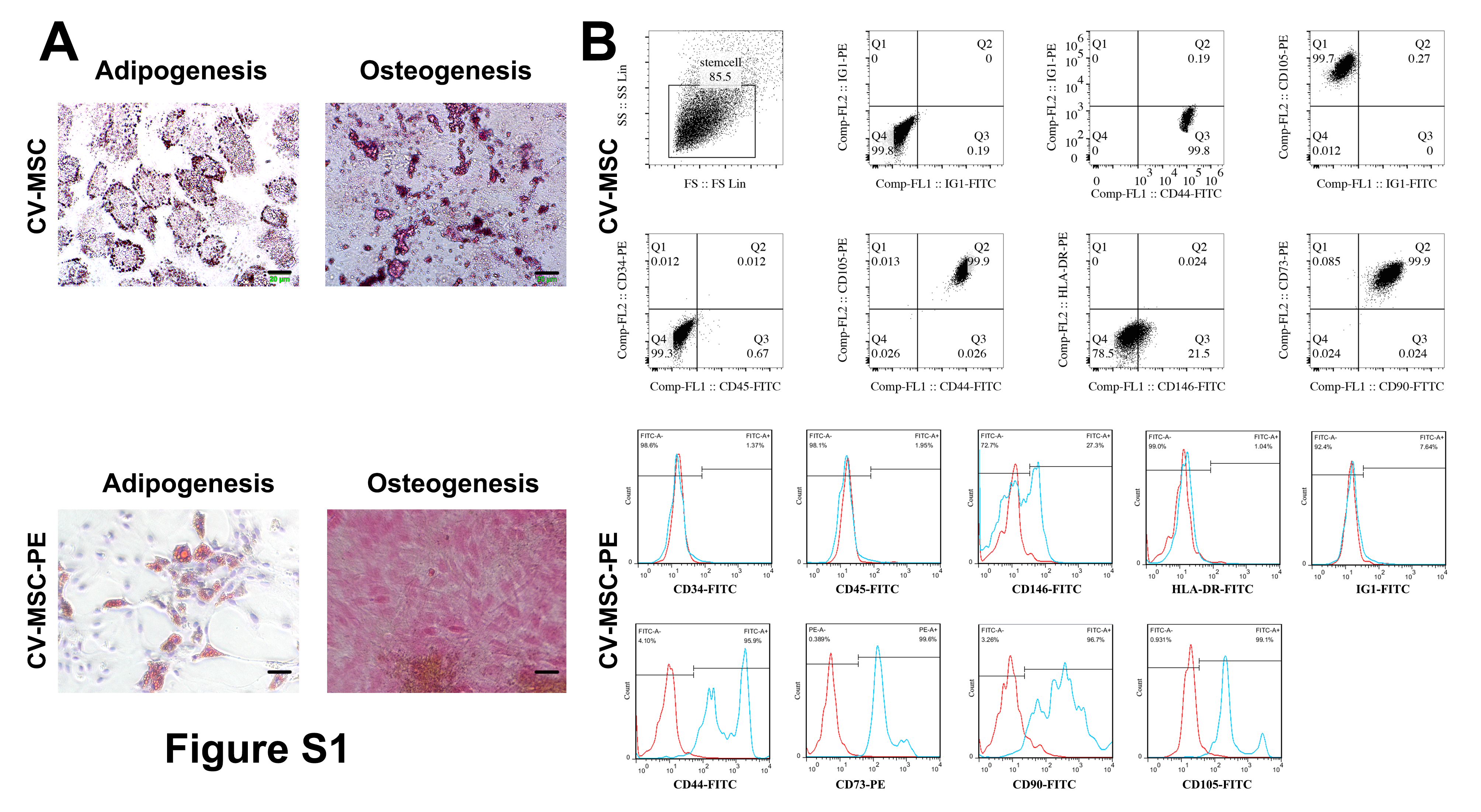

Supplement: Supplementary file 1 — Additional file 1: Figure S1. Characterization of primary CV-MSCs derived from human placental tissues. (A) Representative photomicrographs of primary human CV-MSCs and PE-CV-MSCs before confluence at passage 3. The cells were examined for osteogenic and adipogenic differentiation. Scale bar = 20 μm. (B) The purity of the isolated CV-MSCs and PE-CV-MSCs was examined by flow cytometry; CV-MSCs express CD44, CD73, CD90 and CD105, but lack CD34, CD45, CD146, IG1 and HLA-DR expression. [file 13578_2021_681_MOESM1_ESM.tif]

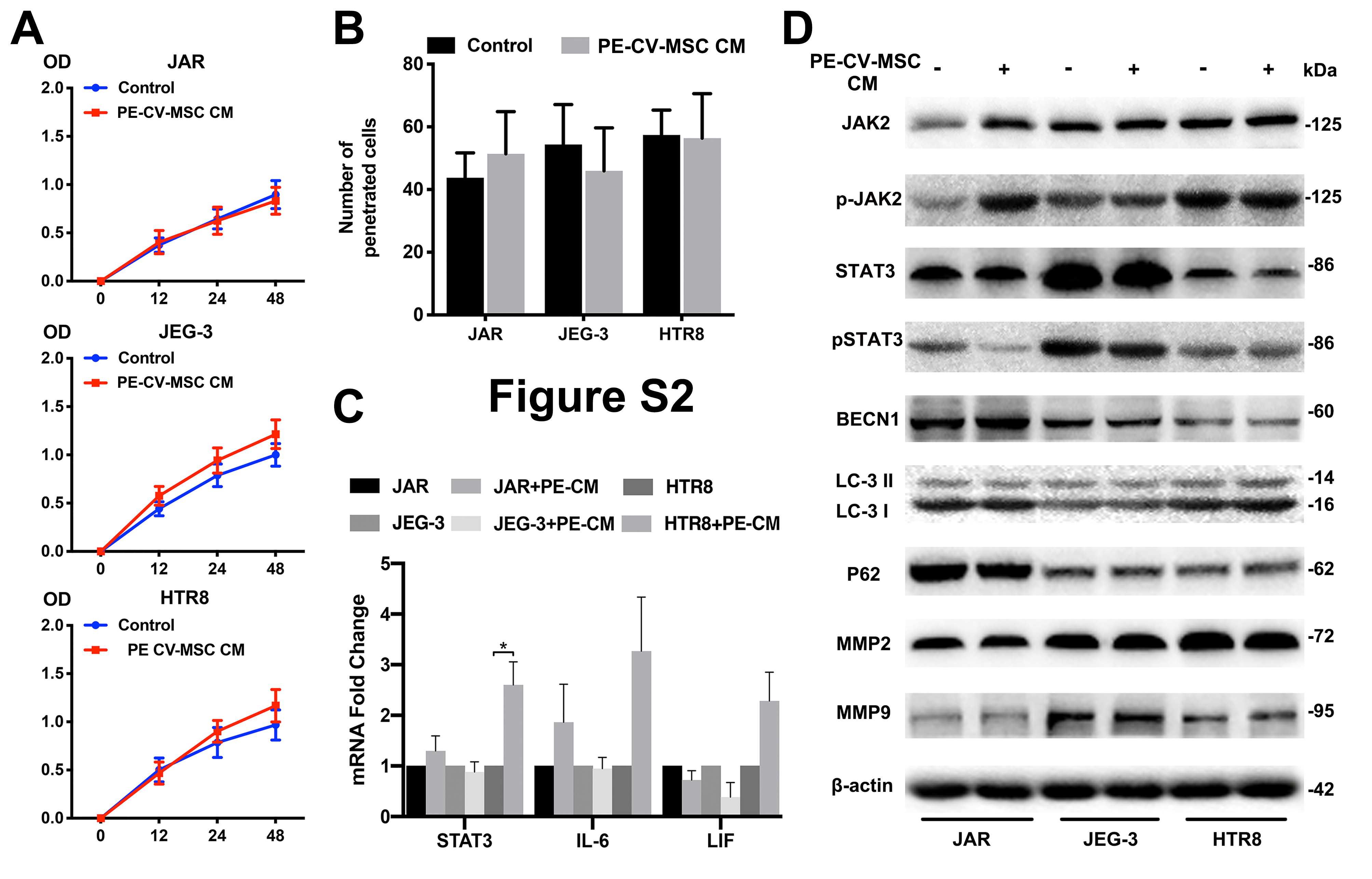

Supplement: Supplementary file 2 — Additional file 2: Figure S2. Effect of PE-CV-MSC CM on the proliferation, invasion, autophagy and JAK2/STAT3 signalling of trophoblasts. (A) Representative CCK-8 assay results in JAR, JEG-3 and HTR-8 cells are shown. Trophoblast cells were treated with PE-CV-MSC CM under hypoxic conditions. (B) The number of trophoblasts that migrated through the 8-μm Transwell membrane pores was counted to determine changes in the invasive capabilities in response to CV-MSC CM under hypoxic conditions. (C) STAT3, IL-6 and LIF mRNA levels were determined in JAR, JEG-3 and HTR-8 cells treated with PE-CV-MSC CM by qRT-PCR. (D) Protein expression of p-JAK2, p-STAT3, LC3 II, BECN1, P62 and MMP2/9 was examined in JAR, JEG-3 and HTR-8 cells treated with PE-CV-MSC CM by western blotting. [file 13578_2021_681_MOESM2_ESM.tif]

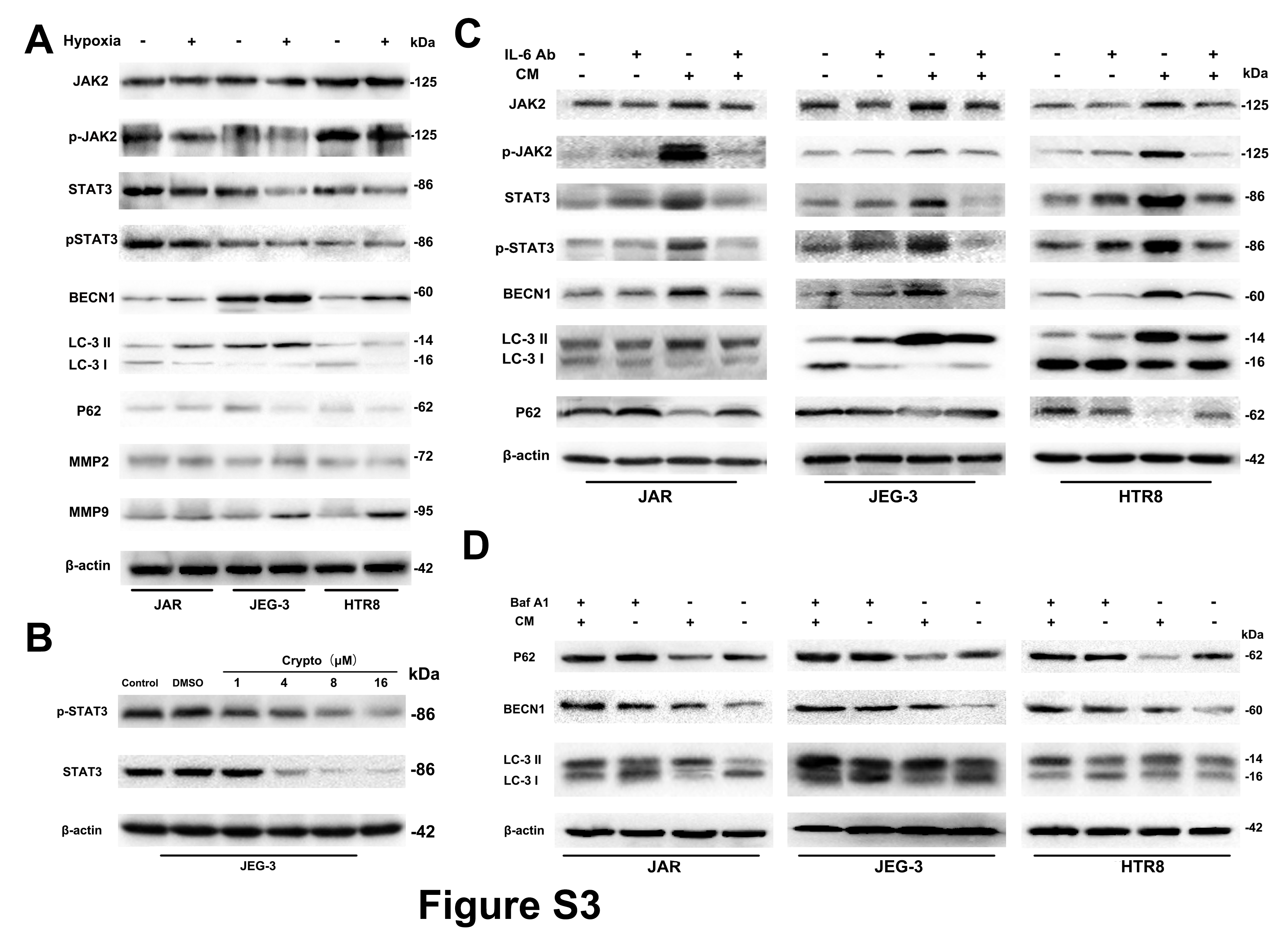

Supplement: Supplementary file 3 — Additional file 3: Figure S3. Effect of hypoxia, IL-6 antibody and Baf A1 on trophoblasts treated with CV-MSC CM. (A) Three trophoblast cell lines were cultured under normal oxygen or hypoxic conditions, and p-JAK2, p-STAT3, BECN1, P62, LC3 and MMP2/9 expression levels was tested by western blotting analysis. (B) STAT3 in JEG-3 cells treated with cryptotanshinone at the indicated concentration or DMSO as a control for 60 min before culture in growth medium were tested by western blotting. (C) IL-6 antibodies were added to trophoblast cells treated with or without CV-MSC CM under hypoxic conditions, and p-JAK2, p-STAT3, BECN1, P62, LC3 and MMP2/9 expression levels were tested by western blotting analysis. Untreated trophoblast cells served as a control. (D) Baf A1 was added to trophoblast cells treated with or without CV-MSC CM under hypoxic conditions, and the expression of BECN1, P62 and LC3 was tested by western blotting analysis. Untreated trophoblast cells served as a control. [file 13578_2021_681_MOESM3_ESM.tif]
